# Who Drives Green Innovation? A Game Theoretical Analysis of a Closed-Loop Supply Chain under Different Power Structures

**DOI:** 10.3390/ijerph17072274

**Published:** 2020-03-27

**Authors:** Dooho Lee

**Affiliations:** Division of Software, Media, and Industrial Engineering, Kangwon National University, 346 Joongang-ro, Samcheok-si, Gangwon-do 29513, Korea; enjdhlee@kangwon.ac.kr

**Keywords:** recycling, green innovation effort, power structure, closed-loop supply chain, game theory

## Abstract

As awareness of environmental protection increases worldwide, enterprises have been building their supply chains in ways that conserve natural resources and minimize the creation of pollutants. One of the practical ways to make supply chains more sustainable is for enterprises to utilize green innovation strategies and to increase resource reuse. In this work, we focus on a closed-loop supply chain (CLSC) consisting of a manufacturer, a retailer, and a collector. In the investigated CLSC, the manufacturer and the retailer drive the green innovation strategy either individually or simultaneously to boost market demand. In the reverse flow of the CLSC, the collector is responsible for collecting consumers’ used products and transferring them to the manufacturer for remanufacturing. By combining two types of the market leadership and three types of green innovation strategies, we establish six different Stackelberg game models and solve them analytically. Through an extensive comparative analysis, we show who should have market leadership and who should drive the green innovation strategy in the CLSC. Various numerical examples are also given to support our major findings. One of our key findings suggests that the supply chain members must participate in green innovation activities at the same time to achieve a win-win scenario in the CLSC.

## 1. Introduction

It has been 33 years since the world began to discuss sustainable development in earnest with the Brundtland Report in 1987. Gro Harlem Brundtland, the Norwegian Prime Minister, chaired the United Nations Conference on the Environment and Development and published the definition of sustainable development in a report entitled *Our Common Future* [1]. According to this report, sustainable development is development that meets the needs of future generations without compromising the ability of future generations to meet their needs. The Brundtland Report included chapters covering, among other topics within sustainable development, the role of the international economy, population and human resources, food security, species and ecosystems, energy, industry, and proposed legal principles for environmental protection [2].

Sustainability also matters in the field of supply chain management (SCM). Over the past few decades, profitability improvement and cost leadership have been the main goals of SCM. However, more recently, the increasing rates of environmental degradation and resource depletion triggered by rapid economic growth have shifted this focus to socio-environmental issues; in the context of supply chain research this has led to greater concern over sustainability, with the concept of supply chain sustainability emerging [3,4,5,6,7]. Consumers’ levels of environmental awareness as well as the implementation of various governmental regulations have forced enterprises in supply chains to employ environmental practices such as green SCM and green innovation. For example, as consumer awareness of environmental protection and sustainable development increases, products with green or ecological labels may become more popular in the market, and the profitability of supply chains may increase [8]. In this context, the concept of green innovation has emerged as a major aspect of sustainable supply chains, with green innovation activities supporting firms in their efforts to become more competitive and engage in sustainable practices in an ever more volatile and highly demanding market [9]. Manufacturers and retailers are willing to improve the degree of the greenness of supply chains through a variety of green innovation activities, such as using clean energy during manufacturing processes, remanufacturing used (or end-of-life) products, developing new technologies to reduce carbon emissions and pollutants, developing new green and sustainable products, and developing new green retailing and marketing techniques. One of the main aims when implementing these green innovation activities is to minimize the impacts of environmental damage while enhancing operational efficiency [10]. In addition, many researchers have pointed out that green product and process innovation applications help companies project a positive corporate image, pioneer new markets, and gain a competitive edge [11,12,13].

With the rapid depletion of resources around the world, waste from used products is becoming an important resource that can be managed globally. Recycling is the process of collecting and processing waste that would otherwise be thrown away as trash and turning the waste into new products for environmental protection. Another aim of recycling is to encourage eco-friendly management and to manage limited supplies of resources. As consumer interest in environmental issues has increased along with the amounts of waste, industrialists and researchers are now focusing on closed-loop supply chains (CLSCs) which are well adapted to sustainability goals [14]. Generally, a CLSC engages in certain operations, such as collecting recyclable waste, transforming it into new materials, and transferring these materials to a manufacturer for remanufacturing. Many countries have been promoting policies related to economic resource circulation. For example, Japan has been encouraging what is termed a sound material-cycle society by implementing the *3R Initiative* (reduce, reuse, recycle) [15]. In fact, green innovation in CLSCs creates many opportunities for closing the recycling loop, from new manufacturing processes for a single product to the creation of a collection-and-processing loop. Recycling ensures that existing resources will be used sustainably. The recycling process alleviates the possibility of the wasteful use of raw materials when they are provided in great abundance. This means that manufacturing industries can prevent existing natural resources from being exploited by the next generation without affecting current production. Moreover, recycling contributes to the reduction of energy consumption, which is vital for large-scale production. Recycling also renders cost savings for supply chain members [16].

Recently, many manufacturers have transferred their dominant positions in supply chains to giant retailers such as Wal-Mart and Amazon to focus on their core business tasks [17,18,19]. As seen in the literature and in the real world, a power shift from manufacturers to retailers in CLSCs can have a major impact on pricing, green innovation efforts, and resource recycling decisions. In this context, the main research questions for this study are as follows:

- Who should drive the green innovation strategy in a CLSC?
- Who should have market leadership in a CLSC?
- How does green innovation strategy and market leadership affect the equilibrium decisions and profits of the members of a CLSC?

The main purpose of this study is to find the answers to these questions. To do this, we consider a CLSC composed of a manufacturer, a retailer, and a collector. After developing six different Stackelberg game models, we demonstrate the existence of equilibrium solutions for each game. By comparing the results of the six game models, we derive significant insights for firms and governments.

The remainder of this paper is organized as follows. In Section 2, we present a review of the relevant literature. In Section 3, we review the notations used and the assumptions made in the paper. In Section 4, the six game models are introduced and solved using game theory. In Section 5, comprehensive comparisons are carried out. In Section 6, we present a summary of the paper and provide future research topics.

## 2. Literature Review

This section deals with the relevant literature considering three different streams of research: green supply chains, recycling issues, and power structures.

### 2.1. Green Supply Chains

Supply chains have significant impacts on the environment through their emissions and pollutants, which affect the health of associated communities. Applying environmental issues to SCM is referred to as green supply chain management (GSCM) [20]. In the literature, many studies have focused on manufacturer’s green innovation strategies. Zhang and Liu [21] examined the coordination mechanism in a three-echelon green supply chain in which the market demand is affected by the greening level of the product. Among the various game models in their study, profits reach their maximal level under cooperative decision-making, whereas they are far from satisfactory in a non-cooperative game. Zhang et al. [22] also revealed that a cooperative game outperforms a non-cooperative game in terms of the profitability of the supply chain in which green and non-green products co-exist and substitute for each other. Madani and Rasti-Barzoki [23] extended the green supply chain to the context of government intervention. In their model, a government aims to encourage supply chains to produce green and sustainable products by presenting subsidies. A numerical experiment conducted by Madani and Rasti-Barzoki [23] showed that as a government provides more subsidies, the greening level of the product becomes higher, which leads to increases in market demand and the profitability of the supply chain. Zhu and He [24] discussed green product design issues in several competition scenarios and insisted that increasing greenness competition hurts the greening level of the product while increasing price competition can be the driving force to increase the greenness of a product. Jamali and Rasti-Barzoki [25] studied the competition between two supply chains in which green products are distributed through one chain and non-green products are distributed through the other. They argued that consumers’ environmental awareness of the green product is a key factor in green and sustainable supply chains. Rahmani and Yavari [26] dealt with pricing and greening decisions for a dual-channel green supply chain considering demand disruptions and found that lower green innovation costs are not only beneficial for the entire supply chain but also increase the greening level of the product.

GSCM research has been so far dominated by studies focusing on manufacturing companies. Recently, studies on retailers’ green innovation strategies have been actively conducted. Nyilasy et al. [27] reported that retailers perceive that green retailing investments have a positive effect on society and the environment and that their investments demonstrate their position as powerful and trustworthy actors for the welfare of the environment. Li et al. [28] analyzed the pricing strategy of a dual-channel supply chain by considering the impacts of green innovations on consumer choices. They concluded that the ability to increase product compatibility with the environment without compromising the quality of products drives retailer’s greening investment decisions. Saha et al. [29] examined the influence of dynamic retailer’s investments in green innovations by considering the reference price effect and argued that the consumer reference price has a significant effect on retailer’s decision to invest in green innovations. Petljak et al. [30] analyzed the relation between green in-store activities and GSCM in food retailing regarding environmental and economic performance. Their data showed that retailer’s greening practices can enhance the environmental and economic performances of supply chains.

Manufacturers and retailers in supply chains may take green innovation strategies at the same time mainly by forming coordination contracts; therefore, coordination contracts are also popular research topics in green supply chains. Song and Gao [31] proved that a revenue-sharing contact can promote the cooperation of upstream and downstream firms and ultimately realize high performance of the green supply chain. Hong and Guo [32] examined several cooperation contracts in a green supply chain and investigated their environmental performance outcomes, showing that a two-part tariff contract leads to the highest greening level of the product and the highest level of cooperation among supply chain members. Tong and Li [33] showed that both government subsidies and greening cost-sharing contracts can achieve the goals of improving the greening level and increasing the market demand level for the green product. Qin et al. [34] also pointed out that participants in green supply chains should cooperate with each other to negotiate a feasible cost-sharing rate.

### 2.2. Recycling and Reusing Issues in Closed-Loop Supply Chains (CLSCs)

The economic and environmental benefits of recycling and reusing have been widely recognized over the past three decades and the CLSC has therefore attracted significant attention from both industry and academia. Through recycling processes, input materials into the CLSC are reduced because some of the generated waste is retrieved to be reused as resources. Thus, the resource dependencies are reduced without affecting economic growth and the CLSC can stimulate the circulation of resources by slowing, narrowing, intensifying and closing resource loops. As Chen et al. [35] pointed out that recycling is one of the major avenues by which to improve the resource utilization. Savaskan and Van Wassenhove [36] studied the reverse channel design and optimal pricing decisions of a CLSC where used products could be collected by two competing recyclers. Chen and Sheu [37] developed a differential game model considering sales competition and recycling dynamics as well as regulation-related profit function. They found that the government must tighten regulatory standards for manufacturers to improve product recycling. Huang et al. [38] studied the effects of recycling competition and showed that dual-channel recycling outperforms single-channel recycling from the perspectives of both manufacturers and consumers. A similar problem with a different used product collection structure was studied by Modak et al. [39], Wang et al. [40], and Liu et al. [41]. Panda et al. [42] developed a socially responsible CLSC with product recycling. They showed that the channel’s non-profit maximizing motive through corporate social responsibility (CSR) practices generates a higher profit margin than the profit maximizing objective. They also suggested that there must be a recycling limit for the best performance of the channel. Shu et al. [43] also studied pricing decisions of CSR CLSCs under carbon cap-and-trade regulations and revealed that the recycling rate is positively affected by firm’s CSR activities and that an increase in the intensity of CSR can lead to reductions in carbon emissions per unit product. He et al. [44] explored recycle pricing strategies in CLSCs considering supply chain members’ fairness concerns and risk-aversion behaviors. Their simulation results showed that when both the manufacturer and retailer are risk-averse, the optimal recycle price in a CLSC achieves its highest level. Li et al. [45] also dealt with the issues of the recycling of construction and demolition waste in CLSCs considering retailer’s fairness concerns. They found that manufacturer’s wholesale price is heavily affected by retailer’s fairness concerns while recycler’s optimal strategy is not affected at all by them.

### 2.3. Power Structures

With the recent emergence of large retailers such as Wal-Mart and Tesco, the power structure in supply chains has had a profound impact on supply chain profitability and sustainability. Therefore, since Spengler [46] first considered the channel structure of supply chains in 1950, many researchers are interested in comparing different channel power structures. Edirisinghe et al. [47] investigated multi-agent supply chains with power imbalances and found that there is no unique supply chain structure that strongly dominates all others. Chen and Zhuang [48] studied the coordination in a retailer-led supply chain consisting of one manufacturer and multiple retailers and proposed that a decentralized supply chain can experience inefficient profit caused by double margination effects. Wang et al. [49] developed a decentralized CLSC which is led by either the manufacturer or the collector. They found that the collector-led CLSC is better at collecting more used products and that such an arrangement decreases environmental damage. Gao et al. [50] explored the impact of different power structures on the equilibrium decisions and profitability of a CLSC. They found that the best power structure for a CLSC varies with the market demand, as influenced by the collection effort. Cheng et al. [51] discussed the relationship between environmental responsibility transfer and market power structure and showed that regardless of who decides upon environmental responsibility transfer, a manufacturer as a game leader may make the environmental and economic performance worse. Gong et al. [19] analyzed the effects of three types of recycling modes in manufacturer-led and retailer-led CLSCs. Their results confirmed that regardless of who has leadership, a hybrid recycling mode outperforms in terms of the overall supply chain profit and recycling performance. Chen et al. [52] analyzed green research and development (R&D) cooperation behavior of firms while considering different power structures. They proved that green R&D cooperation between supply chain members has a positive impact on consumer surplus and environmental protection under retailer-led and manufacturer-led supply chains. Zhang et al. [53] considered cap-and-trade regulations and a green technology strategy by a manufacturer in a supply chain with different power structures. They suggested that an imbalanced power structure helps to reduce carbon emissions. In such a structure, the government must force manufacturers to adopt green technology and set higher carbon caps for them. Liu et al. [54] investigated the influence of power structures and product dual differentiation on pricing decisions in a multi-echelon CLSC and proved that the optimal wholesale, retail, and acquisition prices of a collector-led CLSC are all highest compared to those in manufacturer-led and retailer-led CLSCs. Agi and Yan [55] studied pricing and positioning strategies when green and non-green products are offered in supply chains. They showed that a manufacturer-led supply chain is better prepared than a retailer-led supply chain to overcome fixed costs, launch green products, and identify benefits from the growth of the green consumer segment early in the development stage.

### 2.4. Research Gaps

As seen above, numerous studies have covered green innovation strategies, recycling and reusing issues, and power structures in CLSCs. However, to the best of the author’s knowledge, none of these studies have dealt with the three topics described above simultaneously. Note that green innovation in a CLSC under the different market power structures is a critical question a manufacturer and a retailer often face. With increasing concerns in the environmental protection, supply chain members need an answer to the following question: who should drive green innovation in supply chains? The main contribution of this work is to propose a green innovation strategy that is more beneficial for supply chain members. To do this, we consider three types of green innovation strategies in this paper: manufacturer-driven, retailer-driven, and green innovation strategies driven by both a manufacturer and a retailer. We also aim to investigate and compare the effects of these different power structures on the green innovation, used product collection, and pricing strategies of supply chain members.

## 3. Problem Description

### 3.1. Notations

In this work, we use the notations presented in Table 1.

### 3.2. Assumptions

This work considers a CLSC consisting of one upstream manufacturer (denoted here as “He”), one downstream retailer (“She”), and one third-party collector (“It”). In the forward flow of this CLSC, the manufacturer produces only one type of product and charges the retailer the wholesale price of w. Consumers in the market can purchase their (the manufacturer’s) products at the retail price of p only via the retailer’s channel. The manufacturer makes green (sustainable) innovation investments, such as developing green technology to produce their eco-friendly product. The retailer also invests in green innovation by, for instance, green retailing. Consumers are environmentally conscious and enjoy the helpful environmental attributes of the manufacturer’s and retailer’s green innovation efforts. Regarding the green innovation efforts conducted in the CLSC, we assume three possible green innovation strategies: i) manufacturer-driven green innovation (MG); ii) retailer-driven green innovation (RG); and iii) green innovation driven by both manufactures and retailers (BG). In the MG strategy, only the manufacturer is engaged in green innovation efforts $e_{m}$. Meanwhile, in the RG strategy, only the retailer makes green innovation efforts $e_{r}$. Lastly, in the BG strategy, both the manufacturer and the retailer participate in green innovation with efforts $e_{m}$ and $e_{r}$, respectively.

**Figure 1:**
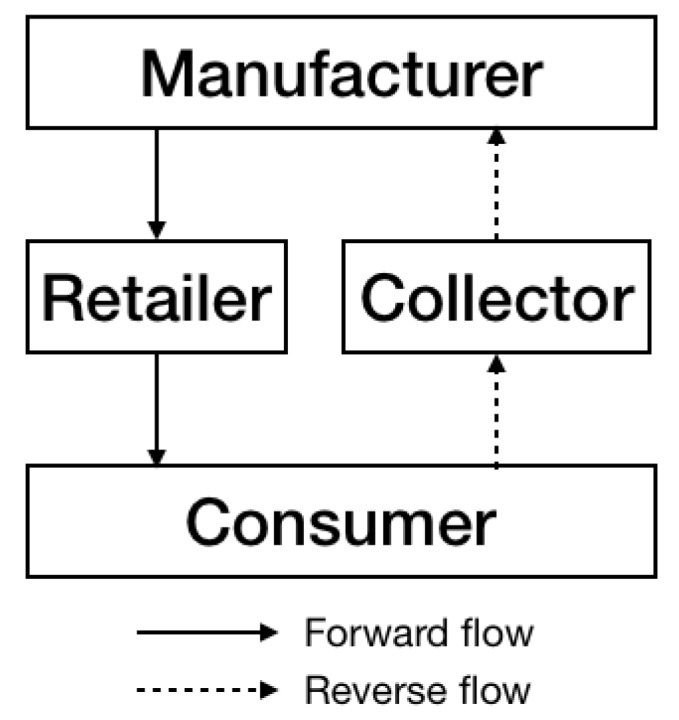
Configuration of the closed-loop supply chain considered in this study.

In the reverse flow of the CLSC, the collector collects the used products that consumers have finished using, after which it transfers the used products to the manufacturer for remanufacturing. In practice, all products that the manufacturer provides to the market cannot be collected. The collecting rate t indicates the portion of used products that can be recovered and turned into a remanufactured product. Figure 1 depicts the configuration of the CLSC considered here. The following assumptions are made:

**Assumption** **1.** *The CLSC considered above is assumed to be based on the make-to-order (MTO) system. In such an MTO system, the order quantity made by the retailer is equal to the production quantity. Thus, none of the supply chain members need to focus on inventory and salvage costs for unsold products caused by demand uncertainties. This work does not consider any stochastic characteristics of the supply chain.*

**Assumption** **2.** *Due to the environment awareness of the consumers, the demand for the product is positively affected by the green innovation efforts. Hence, we use a linear demand function q in Equation (1) to capture the relationship among the demand, retail price, and green innovation efforts.*

(1) $$q=1-p+e_{m}+e_{r}$$

In Equation (1), the potential market base is assumed to be normalized to one. Here, the we assume that $e_{i}$ has a positive coefficient of one; i.e., γ=1. This assumption is justified because the results for an arbitrary γ can be obtained by scaling of the equilibrium green innovation efforts $e_{i}=\gamma {e^{\prime }}_{i}$. Similar assumptions can be found in earlier works, such as those by Chen et al. [56] and Ma et al. [57].

**Assumption** **3.** *We assume that the green innovation efforts affect the unit operation cost $c_{i}=c_{0}+\beta e_{i}$, for i∈{m,r}, with the base cost $c_{0}$ normalized to zero. The symbol β represents a scenario in which green innovation activities by the manufacturer and retailer lead to an increase in the unit operation cost. For example, in the coffee industry, eco-friendly manufacturing processes increase the unit production cost by approximately 30% [56,57,58]. We also restrict our attention to β∈[0,1) such that the supply chain members are assumed to make positive green innovation efforts.*

**Assumption** **4.** *The higher the degree of the green innovation effort, the greater the investment in green innovation development. Thus, the investment in greening by the CLSC considered here is an increasing and convex function of the green innovation efforts, and the green innovation cost is given by $\theta e_{i}^{2}/2$, for i∈{m,r}, where θ is the cost coefficient of the green innovation efforts. In addition, the higher the collecting rate of the used products is, the higher the investment costs associated with the collecting technology will be. According to Gong et al. [19], the collector’s fixed cost is assumed to be $\mu t^{2}/2$, where μ is the cost coefficient of the investment in collecting.*

**Assumption** **5.** *In the reverse flow of the CLSC, the manufacturer buys back the used products that they originally produced from the collector at the transfer price of ρ. The transfer price per unit used product should be greater than the associated acquisition cost α; i.e., ρ>α. The cost saving that the manufacturer realizes by producing remanufactured products is Δ. To encourage the manufacturer to produce remanufactured products, the cost saving Δ is the greater than the transfer price ρ; i.e., Δ>ρ. We use the symbol $K_{1}=(\Delta -\rho )(\rho -\alpha )$. The condition $0<K_{1}<\mu$ is assumed to hold for non-negative decision variables and for optimality of the problem.*

Under Assumptions 1–5, we consider two types of market leadership, i.e., a manufacturer-led CLSC and a retailer-led CLSC, in each of the three green innovation strategies. Therefore, in total, six different Stackelberg games will be analyzed in this paper. The superscript j(j∈Γ={MM,MR,RM,RR,BM,BR}) refers to the name of each game. Table 2 briefly describes the six game models.

## 4. Equilibrium Analyses on Stackelberg Games

In this section, we present the equilibrium decisions of the manufacturer, the retailer, and the collector in each of the six Stackelberg games. All proofs in Section 4 are given Appendix A.

### 4.1. Manufacturer-Driven Green Innovation

First, we discuss the situation in which only the manufacturer drives the green innovation efforts in the CLSC. Therefore, we set $e_{r}=0$. Let $\pi_{m}$, $\pi_{r}$, and $\pi_{c}$ denote the profit functions of the manufacturer, the retailer, and the collector, respectively. $\pi_{m}$, $\pi_{r}$, and $\pi_{c}$ are expressed as follows:

(2) $$\pi_{m}=\left(w-\beta e_{m}\right)q+\left(\Delta -\rho \right)tq-\frac{\theta e_{m}^{2}}{2},\pi_{r}=\left(p-w\right)q,\mathrm{and}\pi_{c}=\left(\rho -\alpha \right)tq-\frac{\mu t^{2}}{2}$$

where $q=1-p+e_{m}$.

#### 4.1.1. Model MM: Manufacturer-Led Stackelberg Game

The sequence of the two-stage Stackelberg game in Model *MM* is as follows. In the first stage of the game, the manufacturer announces the wholesale price w and the degree of their green innovation efforts $e_{m}$. After specifying the values of w and $e_{m}$, in the second stage, the retailer and the collector determine the retail price p and the collecting rate t, respectively. The decisions of the retailer and collector are made simultaneously. Applying backward induction, we can obtain the equilibrium values of the decision variable and the profit for each player in Proposition 1.

**Proposition** **1.** *Assuming that*$2\theta (2\mu -K_{1})>\mu {\left(1-\beta \right)}^{2}$, *the equilibrium values in Model MM are determined as follows:*

(3) $$w^{MM}=\frac{2\theta (\mu -K_{1})+\beta \mu (1-\beta )}{2\theta (2\mu -K_{1})-\mu {\left(1-\beta \right)}^{2}},e_{m}^{MM}=\frac{\mu (1-\beta )}{2\theta (2\mu -K_{1})-\mu {\left(1-\beta \right)}^{2}},\;p^{MM}=1+\frac{\mu (1-\beta -\theta )}{2\theta (2\mu -K_{1})-\mu {\left(1-\beta \right)}^{2}},t^{MM}=\frac{\theta (\rho -\alpha )}{2\theta (2\mu -K_{1})-\mu {\left(1-\beta \right)}^{2}},\;q^{MM}=\frac{\theta \mu }{2\theta (2\mu -K_{1})-\mu {\left(1-\beta \right)}^{2}},\pi_{m}^{MM}=\frac{\theta \mu }{4\theta (2\mu -K_{1})-2\mu {\left(1-\beta \right)}^{2}},\;\pi_{r}^{MM}=\frac{\theta^{2}\mu^{2}}{{\left(2\theta (2\mu -K_{1})-\mu {\left(1-\beta \right)}^{2}\right)}^{2}},\;\mathrm{and}\;\;\pi_{c}^{MM}=\frac{\theta^{2}\mu {\left(\rho -\alpha \right)}^{2}}{2{\left(2\theta (2\mu -K_{1})-\mu {\left(1-\beta \right)}^{2}\right)}^{2}}.$$

#### 4.1.2. Model MR: Retailer-Led Stackelberg Game

The sequence of the two-stage Stackelberg game in Model *MR* is as follows. In the first stage of the game, the retailer announces the retail price. Given the retail price, in the second stage, the manufacturer determines their wholesale price and green innovation efforts while the collector determines its collecting rate. The decisions of the manufacturer and collector are made simultaneously. Backward induction gives the equilibrium values of the decision variable and the profit for each player in Proposition 2.

**Proposition** **2.** *Assuming that*$2\theta >{\left(1-\beta \right)}^{2}$*and*$\theta (2\mu -K_{1})>\mu {\left(1-\beta \right)}^{2}$, *the equilibrium values in Model MR are determined as follows:*

(4) $$w^{MR}=\frac{\theta (\mu -K_{1})+\beta \mu (1-\beta )}{2\theta (2\mu -K_{1})-2\mu {\left(1-\beta \right)}^{2}},e_{m}^{MR}=\frac{\mu (1-\beta )}{2\theta (2\mu -K_{1})-2\mu {\left(1-\beta \right)}^{2}},\;p^{MR}=1+\frac{\mu (1-\beta -\theta )}{2\theta (2\mu -K_{1})-2\mu {\left(1-\beta \right)}^{2}},t^{MR}=\frac{\theta (\rho -\alpha )}{2\theta (2\mu -K_{1})-2\mu {\left(1-\beta \right)}^{2}},\;q^{MR}=\frac{\theta \mu }{2\theta (2\mu -K_{1})-2\mu {\left(1-\beta \right)}^{2}},\pi_{m}^{MR}=\frac{\theta \mu^{2}\left(2\theta -{\left(1-\beta \right)}^{2}\right)}{2{\left(2\theta (2\mu -K_{1})-2\mu {\left(1-\beta \right)}^{2}\right)}^{2}},\;\pi_{r}^{MR}=\frac{\theta \mu }{4\theta (2\mu -K_{1})-4\mu {\left(1-\beta \right)}^{2}},\;\mathrm{and}\;\pi_{c}^{MR}=\frac{\theta^{2}\mu {\left(\rho -\alpha \right)}^{2}}{2{\left(2\theta (2\mu -K_{1})-2\mu {\left(1-\beta \right)}^{2}\right)}^{2}}.$$

#### 4.1.3. Model MM vs. Model MR

We compare the equilibrium values between Model *MM* and Model *MR* in Corollaries 1 and 2.

**Corollary** **1.** $e_{m}^{MR}>e_{m}^{MM}$, $t^{MR}>t^{MM}$*, and*$q^{MR}>q^{MM}$

Corollary 1 shows that when only the manufacturer undertakes green innovations, they make more green innovation efforts if the retailer leads the CLSC. In the retailer-led Stackelberg game, the retailer has leadership on pricing. For the manufacturer, an efficient way to increase their profit would be to undertake more green innovation efforts. As the degree of their green innovation efforts become high, consumers’ utilities increase, which leads to greater demand for the new product. As more new products are sold in the retailer-led game, the number of the used products will increase. Therefore, the collector is likely to collect more used products and transfer them to the manufacturer. That is, the collector enhances its collecting rate in the retailer-led game.

**Corollary** **2.** *We have the following relationships:*

*1. If $\theta <K_{2}$, then $\pi_{m}^{MR}>\pi_{m}^{MM}$; otherwise, $\pi_{m}^{MR}<\pi_{m}^{MM}$;*

*2.* $\pi_{r}^{MR}>\pi_{r}^{MM}$ *and $\pi_{c}^{MR}>\pi_{c}^{MM}$,*

*where* $K_{2}$ *is given in Appendix A.*

**Figure 2:**
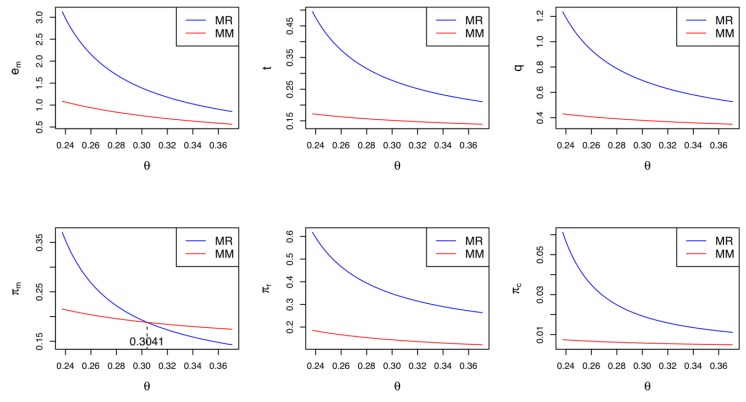
Comparisons between Model *MM* and Model *MR.*

Corollary 2 demonstrates the difference in the supply chain members’ profits between Model *MM* and Model *MR*. We confirm that when only the manufacturer makes green innovation efforts, the retailer and collector prefer the CLSC to be led by the retailer due to their greater profits. Meanwhile, if the cost coefficient of the green innovation efforts is relatively low (i.e., $\theta <K_{2}$), the manufacturer wants the retailer to lead the CLSC; otherwise, they prefer the self-led CLSC. Corollary 2 is consistent with Corollary 1. In Model *MR*, the greater demand and higher collecting rate can improve the profits of the retailer and the collector, respectively. However, the manufacturer’s preferred CLSC depends on the cost sensitivity of their green innovation efforts. Figure 2 illustrates Corollaries 1 and 2 with the following parameter settings: α=0.1, β=0.4, μ=0.5, ρ=0.3, Δ=0.5, $K_{1}=0.04$, and $K_{2}=0.3041$. Varying the value of θ from 0.24 to 0.37, we record the equilibrium results of Model *MM* and Model *MR* in Figure 2. As shown in the figure, in general, Model *MR* is superior to Model *MM*. If $\theta <(>)K_{2}=0.3041$, the manufacturer’s profit in Model *MR* is greater (less) than that in Model *MM*. It is worth noting that the cost coefficient θ has negative impacts on the equilibrium values in both Model *MM* and Model *MR*. This phenomenon is explained as follows: the more cost-sensitive the green innovation is, the fewer green innovation efforts are made. Fewer green innovation efforts result in a decrease in the market demand and collecting rate. Thus, the profits of all supply chain members are reduced.

### 4.2. Retailer-Driven Green Innovation 

Next, we assume a situation in which only the retailer pursues green innovation activities in the CLSC. Setting $e_{m}=0$, the profit functions of the manufacturer, the retailer, and the collector are expressed as follows:

(5) $$\pi_{m}=wq+(\Delta -\rho )tq,\pi_{r}=(p-w-\beta e_{r})q-\frac{\theta e_{r}^{2}}{2},\;\mathrm{and}\;\pi_{c}=(\rho -\alpha )tq-\frac{\mu t^{2}}{2},$$

where $q=1-p+e_{r}$.

#### 4.2.1. Model RM: Manufacturer-Led Stackelberg Game

In Model *RM*, the manufacturer is the leader, and both the retailer and collector are followers. Hence, the sequence of Model *RM* is identical to that of Model *MM*. Applying backward induction, we can obtain the equilibrium values of the decision variable and the profit for each player in Proposition 3.

**Proposition** **3.** *Assuming that $2\theta >{\left(1-\beta \right)}^{2}$ and $\theta (2\mu -K_{1})>\mu {\left(1-\beta \right)}^{2}$, the equilibrium values in Model RM are determined as follows:*

(6) $$w^{RM}=\frac{2\theta (\mu -K_{1})-\mu {\left(1-\beta \right)}^{2}}{2\theta (2\mu -K_{1})-2\mu {\left(1-\beta \right)}^{2}},e_{r}^{RM}=\frac{\mu (1-\beta )}{2\theta (2\mu -K_{1})-2\mu {\left(1-\beta \right)}^{2}},p^{RM}=1+\frac{\mu (1-\beta -\theta )}{2\theta (2\mu -K_{1})-2\mu {\left(1-\beta \right)}^{2}},\;t^{RM}=\frac{\theta (\rho -\alpha )}{2\theta (2\mu -K_{1})-2\mu {\left(1-\beta \right)}^{2}},q^{RM}=\frac{\theta \mu }{2\theta (2\mu -K_{1})-2\mu {\left(1-\beta \right)}^{2}},\pi_{m}^{RM}=\frac{\theta \mu }{4\theta (2\mu -K_{1})-4\mu {\left(1-\beta \right)}^{2}},\;\pi_{r}^{RM}=\frac{\theta \mu^{2}\left(2\theta -{\left(1-\beta \right)}^{2}\right)}{2{\left(2\theta (2\mu -K_{1})-2\mu {\left(1-\beta \right)}^{2}\right)}^{2}},\;\mathrm{and}\;\pi_{c}^{RM}=\frac{\theta^{2}\mu {\left(\rho -\alpha \right)}^{2}}{2{\left(2\theta (2\mu -K_{1})-2\mu {\left(1-\beta \right)}^{2}\right)}^{2}}.$$

#### 4.2.2. Model RR: Retailer-Led Stackelberg Game

In Model *RR*, the retailer is the leader, and both the manufacturer and collector are followers. Thus, the sequence of Model *RR* is identical to that of Model *MR*. Applying backward induction, we derive the equilibrium values of the decision variable and the profit for each player in Proposition 4.

**Proposition** **4.** *Assuming that*$2\theta (2\mu -K_{1})>\mu {\left(1-\beta \right)}^{2}$, *the equilibrium values in Model RR are determined as follows:*

(7) $$w^{RR}=\frac{\theta (\mu -K_{1})}{2\theta (2\mu -K_{1})-\mu {\left(1-\beta \right)}^{2}},e_{r}^{RR}=\frac{\mu (1-\beta )}{2\theta (2\mu -K_{1})-\mu {\left(1-\beta \right)}^{2}},p^{RR}=1+\frac{\mu (1-\beta -\theta )}{2\theta (2\mu -K_{1})-\mu {\left(1-\beta \right)}^{2}},\;t^{RR}=\frac{\theta (\rho -\alpha )}{2\theta (2\mu -K_{1})-\mu {\left(1-\beta \right)}^{2}},q^{RR}=\frac{\theta \mu }{2\theta (2\mu -K_{1})-\mu {\left(1-\beta \right)}^{2}},\pi_{m}^{RR}=\frac{\theta^{2}\mu^{2}}{{\left(2\theta (2\mu -K_{1})-\mu {\left(1-\beta \right)}^{2}\right)}^{2}},\;\pi_{r}^{RR}=\frac{\theta \mu }{2\left(2\theta (2\mu -K_{1})-\mu {\left(1-\beta \right)}^{2}\right)},\;\mathrm{and}\;\pi_{c}^{RR}=\frac{\theta^{2}\mu {\left(\rho -\alpha \right)}^{2}}{2{\left(2\theta (2\mu -K_{1})-\mu {\left(1-\beta \right)}^{2}\right)}^{2}}.$$

#### 4.2.3. Model RM vs. Model RR

We compare the equilibrium values between Model *RM* and Model *RR* in Corollaries 3 and 4.

**Corollary** **3.** $e_{r}^{RM}>e_{r}^{RR}$, $t^{RM}>t^{RR}$*, and*$q^{RM}>q^{RR}$.

Corollary 3 illustrates that when only the retailer drives green innovations, they make more green innovation efforts if the manufacturer leads the CLSC. In the manufacturer-led Stackelberg game, because the manufacturer has leadership on pricing, an efficient way for the retailer to increase their profit would be to conduct more green innovation activities. The more green innovation efforts they make, the higher the consumers’ utilities become, which increases the demand for the new product. According to the increased demand in the manufacturer-led game, the number of the used products will increase. Therefore, the collector is willing to collect more used products and transfer them to the manufacturer. That is, the collector enhances its collecting rate in the manufacturer-led game.

**Corollary** **4.** *We have the following relationships:*

*1. If $\theta <K_{2}$, then $\pi_{r}^{RM}>\pi_{r}^{RR}$; otherwise, $\pi_{r}^{RM}<\pi_{r}^{RR}$;*

*2.* $\pi_{m}^{RM}>\pi_{m}^{RR}$ *and $\pi_{c}^{RM}>\pi_{c}^{RR}$.*

**Figure 3:**
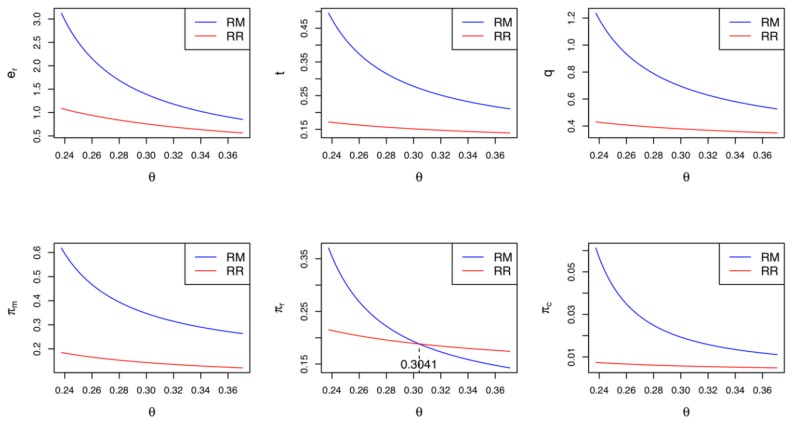
Comparisons between Model *RR* and Model *RM.*

The statements in Corollary 4 are similar to those in Corollary 2 above. When only the retailer engages in the green innovation, the manufacturer and the collector prefer the CLSC to be led by the manufacturer due to their greater profits. Meanwhile, if the cost coefficient of the green innovation efforts is relatively low (i.e., $\theta <K_{2}$), the retailer wants the manufacturer to lead the CLSC; otherwise, they prefer a self-led CLSC. Corollary 4 is consistent with Corollary 2. In Model *RM*, the greater demand and higher collecting rate can improve the profits of the manufacturer and the collector, respectively. However, the retailer’s preferred CLSC depends on the cost sensitivity of the green innovation efforts they make. Summarizing Corollaries 1–4, we can infer one of the key findings: if the manufacturer (retailer) utilizes a green innovation strategy and the green innovation cost is relatively low, the market leadership is better if given to the retailer (manufacturer) in terms of the profitability and environmental aspects of the CLSC. Figure 3 depicts Corollaries 3 and 4 with parameters identical to those in Figure 2. It should be noted that if $\theta <(>)K_{2}=0.3041$, the retailer’s profit in Model *RM* is greater (less) than that in Model *RR*. The rest of Figure 3 is quite similar to Figure 2; therefore, we omit further statements.

### 4.3. Both-Driven Green Innovation 

Next, we assume a situation in which both the manufacturer and the retailer make green innovation efforts in the CLSC. The profit functions of the manufacturer, the retailer, and the collector then are written as follows:

(8) $$\pi_{m}=(w-\beta e_{m})q+(\Delta -\rho )tq-\frac{\theta e_{m}^{2}}{2},\pi_{r}=(p-w-\beta e_{r})q-\frac{\theta e_{r}^{2}}{2},\;\mathrm{and}\;\pi_{c}=(\rho -\alpha )tq-\frac{\mu t^{2}}{2},$$

where $q=1-p+e_{m}+e_{r}$.

#### 4.3.1. Model BM: Manufacturer-Led Stackelberg Game

In Model *BM*, the manufacturer is the leader, and both the retailer and the collector are followers. Hence, the sequence of Model *BM* is identical to that of Model *MM*. Applying backward induction, we can obtain the equilibrium values of the decision variable and the profit for each player in Proposition 5.

**Proposition** **5.** *Assuming that*$2\theta >{\left(1-\beta \right)}^{2}$, $\theta (2\mu -K_{1})>\mu {\left(1-\beta \right)}^{2}$*, and $2\theta (2\mu -K_{1})>3\mu {\left(1-\beta \right)}^{2}$, the equilibrium values in Model BM are determined as follows:*

(9) $$w^{BM}=\frac{2\theta (\mu -K_{1})-\mu (1-\beta )(1-2\beta )}{2\theta (2\mu -K_{1})-3\mu {\left(1-\beta \right)}^{2}},e_{m}^{BM}=\frac{\mu (1-\beta )}{2\theta (2\mu -K_{1})-3\mu {\left(1-\beta \right)}^{2}},\;p^{BM}=1+\frac{\mu (2-2\beta -\theta )}{2\theta (2\mu -K_{1})-3\mu {\left(1-\beta \right)}^{2}},e_{r}^{BM}=\frac{\mu (1-\beta )}{2\theta (2\mu -K_{1})-3\mu {\left(1-\beta \right)}^{2}},t^{BM}=\frac{\theta (\rho -\alpha )}{2\theta (2\mu -K_{1})-3\mu {\left(1-\beta \right)}^{2}},\;q^{BM}=\frac{\theta \mu }{2\theta (2\mu -K_{1})-3\mu {\left(1-\beta \right)}^{2}},\pi_{m}^{BM}=\frac{\theta \mu }{2\left(2\theta (2\mu -K_{1})-3\mu {\left(1-\beta \right)}^{2}\right)},\;\pi_{r}^{BM}=\frac{\theta \mu^{2}\left(2\theta -{\left(1-\beta \right)}^{2}\right)}{2{\left(2\theta (2\mu -K_{1})-3\mu {\left(1-\beta \right)}^{2}\right)}^{2}},\;\mathrm{and}\;\pi_{c}^{BM}=\frac{\theta^{2}\mu {\left(\rho -\alpha \right)}^{2}}{2{\left(2\theta (2\mu -K_{1})-3\mu {\left(1-\beta \right)}^{2}\right)}^{2}}.$$

#### 4.3.2. Model BR: Retailer-Led Stackelberg Game

In Model *BR*, the retailer is the leader, and both the manufacturer and the collector are followers. Hence, the sequence of Model *BR* is identical to that of Model *MR*. Applying backward induction, we can obtain the equilibrium values of the decision variable and the profit for each player in Proposition 6.

**Proposition** **6.** *Assuming that $2\theta >{\left(1-\beta \right)}^{2}$, $\theta (2\mu -K_{1})>\mu {\left(1-\beta \right)}^{2}$, and $2\theta (2\mu -K_{1})>3\mu {\left(1-\beta \right)}^{2}$, the equilibrium values in Model BM are determined as follows:*

(10) $$w^{BR}=\frac{2\theta (\mu -K_{1})+\mu \beta (1-\beta )}{2\theta (2\mu -K_{1})-3\mu {\left(1-\beta \right)}^{2}},e_{m}^{BR}=\frac{\mu (1-\beta )}{2\theta (2\mu -K_{1})-3\mu {\left(1-\beta \right)}^{2}},p^{BR}=1+\frac{\mu (2-2\beta -\theta )}{2\theta (2\mu -K_{1})-3\mu {\left(1-\beta \right)}^{2}}\;,e_{r}^{BR}=\frac{\mu (1-\beta )}{2\theta (2\mu -K_{1})-3\mu {\left(1-\beta \right)}^{2}},t^{BR}=\frac{\theta (\rho -\alpha )}{2\theta (2\mu -K_{1})-3\mu {\left(1-\beta \right)}^{2}},q^{BR}=\frac{\theta \mu }{2\theta (2\mu -K_{1})-3\mu {\left(1-\beta \right)}^{2}},\;\pi_{m}^{BR}=\frac{\theta \mu^{2}\left(2\theta -{\left(1-\beta \right)}^{2}\right)}{2{\left(2\theta (2\mu -K_{1})-3\mu {\left(1-\beta \right)}^{2}\right)}^{2}},\pi_{r}^{BR}=\frac{\theta \mu }{2\left(2\theta (2\mu -K_{1})-3\mu {\left(1-\beta \right)}^{2}\right)},\;\mathrm{and}\;\;\pi_{c}^{BR}=\frac{\theta^{2}\mu {\left(\rho -\alpha \right)}^{2}}{2{\left(2\theta (2\mu -K_{1})-3\mu {\left(1-\beta \right)}^{2}\right)}^{2}}.$$

#### 4.3.3. Model BM vs. Model BR

We compare the equilibrium values between Model *BM* and Model *BR* in Corollaries 5 and 6.

**Corollary** **5.** *$e_{m}^{BM}=e_{m}^{BR}=e_{r}^{BM}=e_{r}^{BR}$, $t^{BM}=t^{BR}$, and $q^{BM}=q^{BR}$.*

Corollary 5 shows that when both the manufacturer and retailer participate in green innovation activities, they maintain the same degree of green innovation effort regardless of who leads the CLSC. As the level of demand is directly influenced by the green innovation efforts and by the price of the product, the demand for the product in both the manufacturer-led CLSC and the retailer-led CLSC remains the same. In both game models, the demands are identical; hence, the collector’s collecting rates are also identical.

**Corollary** **6.** *We have the following relationships:*

*1. If $\theta <K_{3}$, then $\pi_{m}^{BM}<\pi_{m}^{BR}$ and $\pi_{r}^{BM}>\pi_{r}^{BR}$;*

*2. if $\theta >K_{3}$, then $\pi_{m}^{BM}>\pi_{m}^{BR}$ and $\pi_{r}^{BM}<\pi_{r}^{BR}$;*

*3. $\pi_{c}^{BM}=\pi_{c}^{BR}$,*

*where $K_{3}=\mu {\left(1-\beta \right)}^{2}/(\mu -K_{1})$.*

**Figure 4:**
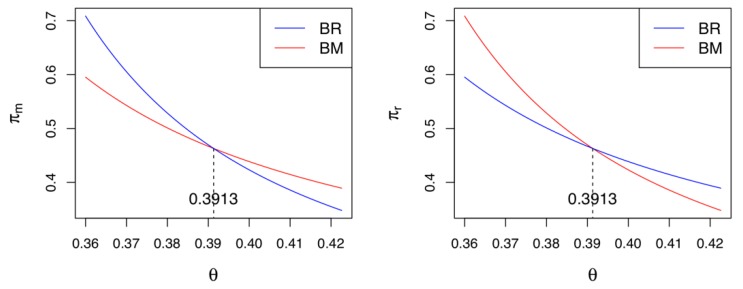
Comparisons between Model *BR* and Model *BM.*

Corollary 6 reveals that when both the manufacturer and retailer participate in green innovation activities, if the cost coefficient of the green innovation is relatively low (i.e., $\theta <K_{3}$), the manufacturer (retailer) prefers the CLSC to be led by the retailer (manufacturer). Otherwise, they prefer a self-led CLSC. This finding implies that when the manufacturer and retailer make green innovation efforts at the same time, their preferred CLSC depends on their green innovation costs. Note that from the collector’s perspective, it does not matter who leads the CLSC because its profits are identical in either case. Figure 4 presents the equilibrium profits of the manufacturer and the retailer with the same parameter settings used above. If $\theta <K_{3}=0.3913$, the manufacturer (retailer) prefers a retailer-led (manufacturer-led) CLSC; otherwise, both the manufacturer and the retailer prefer a self-led CLSC.

## 5. Comparative Analysis

In this section, comprehensive comparisons are carried out to obtain the significant findings of the paper. All proofs in Section 5 are found in Appendix B.

### 5.1. Comparison of Green Innovation Efforts, Collecting Rates, and Demands among Different Models

**Corollary** **7.** *Let $e_{t}^{j}$, for j∈Γ, be the total green innovation efforts made in the CLSC. We have the following relationships:*

*1. $e_{t}^{BM}=e_{t}^{BR}>e_{t}^{MR}=e_{t}^{RM}>e_{t}^{MM}=e_{t}^{RR}$;*

*2. $t^{BM}=t^{BR}>t^{MR}=t^{RM}>t^{MM}=t^{RR}$;*

*3. $q^{BM}=q^{BR}>q^{MR}=q^{RM}>q^{MM}=q^{RR}$.*

**Figure 5:**
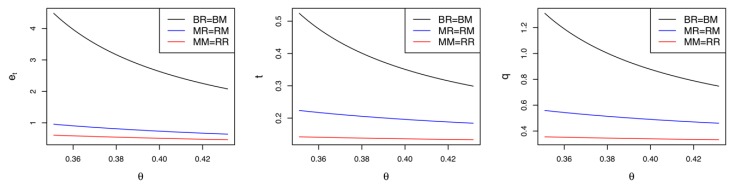
Comparisons of the green innovation effort, the collecting rate, and the market demand among the six game models.

Corollary 7 shows that Models *BM* and *BR*, the CLSCs in which the manufacturer and the retailer respectively drive green innovation simultaneously, achieve the highest degree of green innovation effort, the highest collecting rate, and the greatest levels of market demand. On the other hand, Models *MM* and *RR*, correspondingly the CLSCs in which only the market leader engages in green innovation, have the lowest degree of green innovation effort, the lowest collecting rate, and the lowest level of market demand. Models *MR* and *RM*, the CLSCs in which only the market follower engages in the green innovation effort, are ranked in the middle. Therefore, we can conclude that regardless of who leads the CLSC, the BG strategy is the optimal approach in order to realize the greatest levels of demand and green innovation effort while also offering the highest collecting rate to the collector. In Figure 5, we can find the results in Corollary 7. That is, Models *BM* and *BR* outperform any of the other game models in terms of green innovation effort, the collecting rate, and market demand. Meanwhile, the performances of Models *MM* and *RR* are the poorest among the six game models.

### 5.2. Comparison of Profits among Different Models Led by the Manufacturer

**Corollary** **8.** *$\pi_{m}^{BM}>\pi_{m}^{RM}>\pi_{m}^{MM}$, $\pi_{r}^{BM}>\pi_{r}^{RM}>\pi_{r}^{MM}$, and $\pi_{c}^{BM}>\pi_{c}^{RM}>\pi_{c}^{MM}$.*

Corollary 8 reveals that in the manufacturer-led CLSC, the BG strategy guarantees the highest profits for all supply chain members, while the MG strategy provides the lowest. The RG strategy is located between the BG and MG strategies. Thus, when market leadership is given to the manufacturer, green innovation efforts should be made by both the manufacturer and the retailer in order to achieve the highest profits.

### 5.3. Comparison of Profits among Different Models Led by the Retailer

**Corollary** **9.** *$\pi_{m}^{BR}>\pi_{m}^{MR}>\pi_{m}^{RR}$, $\pi_{r}^{BR}>\pi_{r}^{MR}>\pi_{r}^{RR}$ and $\pi_{c}^{BR}>\pi_{c}^{MR}>\pi_{c}^{RR}$.*

**Figure 6:**
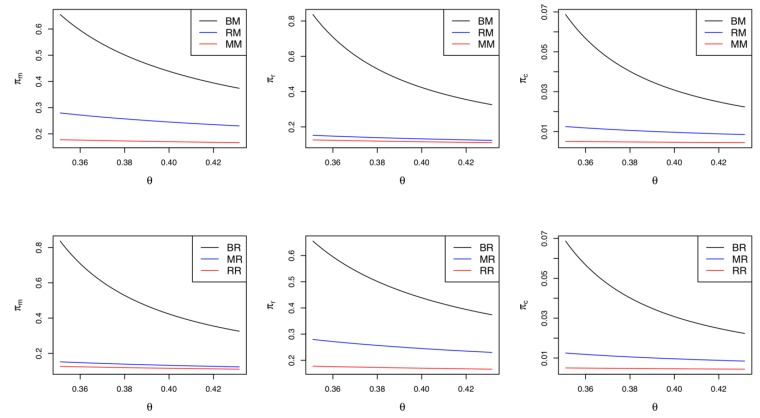
Comparisons of profits in the closed-loop supply chains (CLSC) led by the manufacturer and the retailer.

The results in Corollary 9 are similar to those in Corollary 8. In the retailer-led CLSC, the BG strategy also guarantees the highest profit for all supply chain members, while the RG strategy provides the lowest. Corollaries 8 and 9 are consistent with Corollary 7. That is, the stronger the green innovation efforts and the higher the collecting rate of used products in the CLSC, the higher the demand for the product and ultimately the higher the profitability of the supply chain members. Summarizing Corollaries 8 and 9, regardless of who leads the market, both the manufacturer and the retailer must make green innovation efforts at the same time to ensure the highest profits for the supply chain members. Figure 6 is a numerical example of Corollaries 8 and 9. We can also find that Models *BM* and *BR* outperform any of the other game models in terms of the profitability of the CLSC, while the Models *MM* and *RR* results in the lowest profits.

### 5.4. Comparison of Profits among Models MM, MR, RM, and RR

**Corollary** **10.** *We have the following relationships:*

*1. If $\theta <K_{3}/2$, then $\pi_{m}^{MR}>\max\left\{\pi_{m}^{RM},\pi_{m}^{MM},\pi_{m}^{RR}\right\}$; otherwise, $\pi_{m}^{RM}>\max\left\{\pi_{m}^{MR},\pi_{m}^{MM},\pi_{m}^{RR}\right\}$;*

*2. if $\theta <K_{3}/2$, then $\pi_{r}^{RM}>\max\left\{\pi_{r}^{MR},\pi_{r}^{MM},\pi_{r}^{RR}\right\}$; otherwise, $\pi_{r}^{MR}>\max\left\{\pi_{r}^{RM},\pi_{r}^{MM},\pi_{r}^{RR}\right\}$;*

*3. if* $\theta <K_{3}/2$ *, then* $\pi_{m}^{MM}<\min\left\{\pi_{m}^{MR},\pi_{m}^{RM},\pi_{m}^{RR}\right\}$ *; otherwise, $\pi_{m}^{RR}<\min\left\{\pi_{m}^{MR},\pi_{m}^{RM},\pi_{m}^{MM}\right\}$;*

*4. if $\theta <K_{3}/2$, then $\pi_{r}^{RR}<\min\left\{\pi_{r}^{MR},\pi_{r}^{RM},\pi_{r}^{MM}\right\}$; otherwise, $\pi_{r}^{MM}<\min\left\{\pi_{r}^{MR},\pi_{r}^{RM},\pi_{r}^{RR}\right\}$;*

*5. $\pi_{c}^{MR}=\pi_{c}^{RM}>\pi_{c}^{MM}=\pi_{c}^{RR}$.*

**Figure 7:**
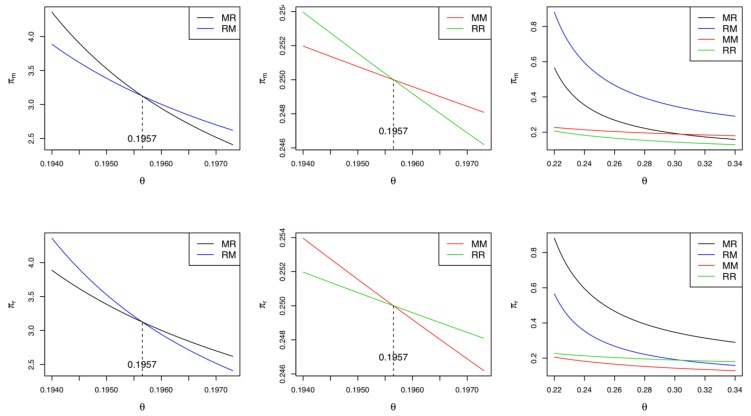
Comparisons of the profits when single supply chain member makes the green innovation efforts.

Corollary 10 compares the profits among Models *MM*, *MR*, *RM*, and *RR*. Note that only one between the manufacturer and the retailer drives green innovation in these models. As stated in Corollary 10 and displayed in Figure 7, if the cost coefficient of green innovation is relatively low (i.e., $\theta <K_{3}/2=0.1957$), the manufacturer’s most preferred situation is Model *MR*, where market leadership is given to the retailer and green innovation efforts are only made by the manufacturer. In contrast, if the cost coefficient of green innovation is relatively high (i.e., $\theta >K_{3}/2$), their most preferred situation changes from Model *MR* to Model *RM*, where market leadership is theirs and the retailer undertakes green innovation efforts. Moreover, if $\theta <K_{3}/2$, the manufacturer’s least preferred situation is Model *MM*, where they drive green innovations and lead the CLSC. Otherwise, their least preferred situation changes to Model *RR*, where the retailer is in charge of both green innovations and market leadership. With regard to the retailer, the situation is opposite that of the manufacturer. If $\theta <K_{3}/2$ ($\theta >K_{3}/2$), the retailer’s most preferred situation is Model *RM* (Model *MR*); otherwise, their least preferred situation is Model *RR* (Model *MM*). Summarizing Corollary 10, the optimal situation for the manufacturer and the retailer depends on who oversees green innovations and who leads the market. From the collector’s point of view, Models *MR* and *RM* lead to identical profits, and these models are superior to Models *MM* and *RR.* That is, the collector always prefers a situation in which green innovations and market leadership are left to different players. The collector’s numerical example is presented in Figure 6.

### 5.5. Comparison of Profits among All Six Models

**Corollary** **11.** *We have the following relationships:*

*1. If $K_{4}<\theta <K_{3}$, then $\pi_{m}^{BR}>\max\left\{\pi_{m}^{BM},\pi_{m}^{RM},\pi_{m}^{MR},\pi_{m}^{MM},\pi_{m}^{RR}\right\}$ and $\pi_{r}^{BM}>\max\left\{\pi_{r}^{BR},\pi_{r}^{RM},\pi_{r}^{MR},\pi_{r}^{MM},\pi_{r}^{RR}\right\}$;*

*2. if* $\theta >K_{3}$ *, then* $\pi_{m}^{BM}>\max\left\{\pi_{m}^{BR},\pi_{m}^{RM},\pi_{m}^{MR},\pi_{m}^{MM},\pi_{m}^{RR}\right\}$ *and $\pi_{r}^{BR}>\max\left\{\pi_{r}^{BM},\pi_{r}^{RM},\pi_{r}^{MR},\pi_{r}^{MM},\pi_{r}^{RR}\right\}$;*

*3. $\pi_{m}^{RR}<\min\left\{\pi_{m}^{BM},\pi_{m}^{BR},\pi_{m}^{RM},\pi_{m}^{MR},\pi_{m}^{MM}\right\}$ and $\pi_{r}^{MM}<\min\left\{\pi_{r}^{BM},\pi_{r}^{BR},\pi_{r}^{RM},\pi_{r}^{MR},\pi_{r}^{RR}\right\}$;*

*4. $\pi_{c}^{BM}=\pi_{c}^{BR}>\pi_{c}^{RM}=\pi_{c}^{MR}>\pi_{c}^{MM}=\pi_{c}^{RR}$,*

*where $K_{4}=3\mu {\left(1-\beta \right)}^{2}/2(2\mu -K_{1})$.*

**Figure 8:**
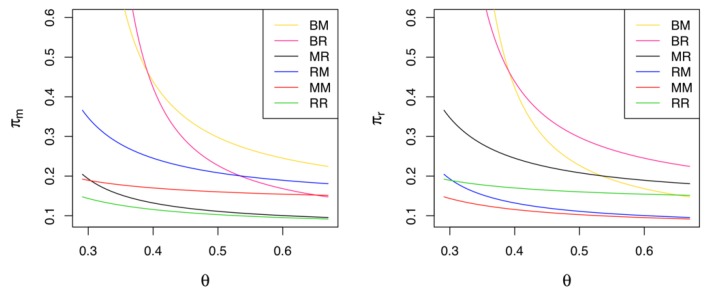
Comparisons of profits among all six models.

Corollary 11 states that the most and least preferred game models also depend on the value of θ. Note that in order to compare the profits among all six models, the optimality condition should satisfy the condition of $\theta >K_{4}$. As stated in Corollary 11 and shown in Figure 8, if the cost coefficient of green innovation is relatively low (i.e., $\theta <K_{3}=0.3913$), the manufacturer’s and retailer’s most preferred situations are Model *BR* and *BM*, respectively. In contrast, if the cost coefficient is relatively high (i.e., $\theta >K_{3}$), the most preferred models for the manufacturer and the retailer change to Model *BM* and Model *BR*, respectively. Regardless of who leads the CLSC, a situation in which the manufacturer and the retailer undertake green innovation efforts at the same time ensures the highest profits for them. In addition, the manufacturer’s (retailer’s) worst situation is Model *RR* (Model *MM*), in which the retailer (the manufacturer) is responsible for green innovations and they also have market leadership. For the collector, its most preferred situations are Models *BM* and *BR* while its least ones are Models *MM* and *RR*. The collector’s numerical example is also presented in Figure 6.

### 5.6. Comparison of Supply Chain Profits among Different Models

**Corollary** **12.** *Let* $\pi_{sc}^{j}$ *, for* j∈Γ *, be the total profit in the CLSC (i.e.,* $\pi_{sc}^{j}=\pi_{m}^{j}+\pi_{r}^{j}+\pi_{c}^{j}$ *). We have the following relationship: $\pi_{sc}^{BM}=\pi_{sc}^{BR}>\pi_{sc}^{RM}=\pi_{sc}^{MR}>\pi_{sc}^{MM}=\pi_{sc}^{RR}$.*

**Figure 9:**
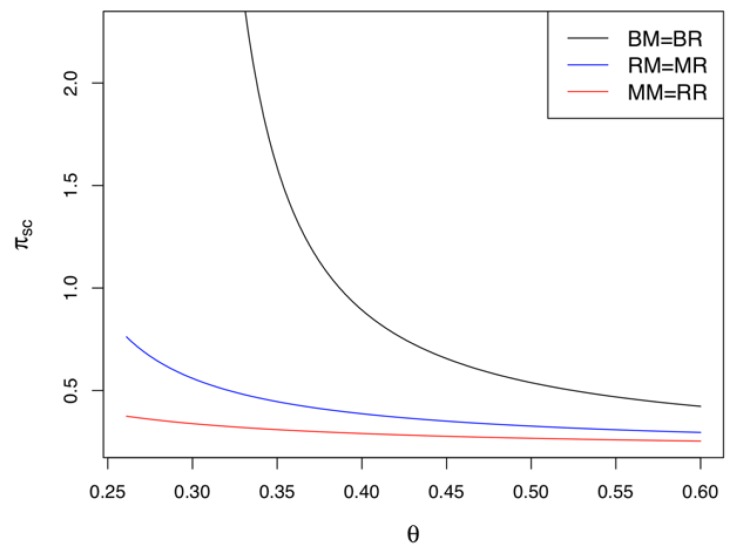
Comparisons of the supply chain profits among different models.

Corollary 12 shows that in terms of the profitability of the CLSC, the BG strategy outperforms the MG and RG strategies. That is, regardless of who leads the CLSC, game models in which the manufacturer and the retailer participate in green innovation activities simultaneously are more profitable than any of the other game models. Meanwhile, Models *MM* and *RR*, in which the market leader makes green innovation efforts, are the least profitable situations. This matches our findings in Corollary 7. From the perspectives of both individual players and the entire system, the BG strategy is best with regard to more green innovation efforts, the collection of more used products, and the boosting of market demand; it can not only maximize supply chain profits but also encourage the supply chain members to focus on environmental protection concepts, such as green innovations and recycling. Once again, to realize a win-win situation in the CLSC, both the manufacturer and retailer should adopt the BG strategy. For both the retailer and manufacturer to undertake a green innovation strategy, the government must provide monetary incentives, including subsidies and/or tax exemptions for green innovation efforts. Figure 9 shows why governmental financial support for green innovations should be provided.

## 6. Future Research Topics

This work provides certain recommendations for firms that make greening and collecting efforts in various power structures. However, there are also new areas of improvements which can be analyzed in future research. (1) Our demand functions do not consider any stochastic features; however, the assumption of uncertain demand for the product is more realistic. (2) In the game models considered here, the collector makes no green innovation efforts. A possible extended model can assume that the collector also jointly participates in green innovations. This assumption may lead to better profits for the supply chain members. (3) A variety of the supply chain contracts, including the cost-sharing, revenue-sharing, and two-part tariff types, as well as put option contracts may encourage supply chain members to collaborate with each other and improve the sustainability and the profitability of the supply chain. (4) This work did not consider any competition in the CLSC. However, in the real world, multiple manufacturers, retailers, and collectors collaborate/compete with each other. It would be valuable to analyze CLSCs with more complex but realistic conditions.

## 7. Conclusions

This study investigated the equilibrium pricing, green innovation, and collecting decisions in the CLSC composed of a manufacturer, a retailer, and a collector. It was assumed that the CLSC takes three types of the green innovation strategy, namely Strategy MG, Strategy RG, and Strategy BG. In each green innovation strategy, two types of the market leadership, namely manufacturer-led and retailer-led CLSCs, were considered. Thus, in total, six different game models were developed and analyzed in detail using the Stackelberg game framework. Extensive numerical examples were provided to support our findings. Our major findings are summarized below.

- Under the MG strategy, where only the manufacturer drives green innovations, the retailer and the collector always prefer a retailer-led CLSC. Meanwhile, the manufacturer prefers a self-led CLSC (retailer-led CLSC) if the cost sensitivity of the green innovation efforts is high (low).
- Under the RG strategy, where only the retailer drives green innovations, the manufacturer and the collector always prefer a manufacturer-led CLSC. Meanwhile, the retailer prefers a self-led CLSC (manufacturer-led CLSC) if the cost sensitivity of the green innovation efforts is high (low).
- Under the BG strategy, where both the manufacturer and retailer are in charge of green innovations, the profits for the collector are identical in both a manufacturer-led and a retailer-led CLSC. If the cost sensitivity of the green innovation efforts is relatively low, the manufacturer (retailer) prefers a CLSC led by the retailer (manufacturer). Otherwise, they prefer self-led CLSCs.
- In the manufacturer-led CLSC, all supply chain members prefer the BG strategy the most and the MG strategy the least. Similarly, in the retailer-led CLSC, all supply chain members also prefer the BG strategy the most and the RG strategy the least.
- When only one member drives green innovations in the CLSC, the most and least profitable game model for each supply chain member will depend on the cost of the green innovation efforts.
- If the cost sensitivity of the green innovation efforts is relatively low (high), among the six different game models analyzed here, the manufacturer achieves the highest profit in Model BR (Model BM), where the retailer-led (manufacturer-led) CLSC uses the BG strategy. Similarly, if it is relatively low (high), the retailer achieves the highest profits in Model BM (Model BR). The collector’s most profitable games are Models BM and BR, where its profits are identical.
- Under the BG strategy, the overall green innovation efforts, the collecting rate of used products, and the market demand are all highest. Therefore, the BG strategy not only maximizes the profits of the supply chain but also encourages its members to focus on environmental protection issues, such as green innovations and recycling.

From the results summarized above, several managerial and administrative insights can be derived for firms and governments. Overall, the BG strategy is more profitable than the other strategies. The similar result can be found in Gong et al. [19], where authors emphasized that it is always advantageous for manufacturers and retailers to participate in recycling at the same time in terms of the sustainability and profitability of CLSCs. Hence, the manufacturer and retailer are expected to benefit even more from a cooperative effort when implementing green innovation strategies. Many researchers have proved that the elimination of the double-margination effect via cooperation among supply chain members can improve the profitability as well as the sustainability of the supply chain. Accordingly, policymakers must devise a variety of monetary benefit policies, such as subsidies and/or tax exemptions, to encourage such cooperation in green innovations by members of supply chain. The literature on green innovations in supply chains argues that governmental subsidies for environmentally friendly activities can ensure stable and sustainable supply chains.

## Figures and Tables

**Table 1:** Notations.

**Parameters**	**Descriptions**
α β θ μ ρ Δ	Acquisition cost of a used product incurred by the recycler Change in operation cost due to green innovation effort Cost coefficient of green innovation efforts Collecting investment coefficient Transfer price of a used product paid by the manufacturer Savings per unit from remanufacturing
**Decision variables**	**Descriptions**
$e_{m}$ $e_{r}$ p t w	Green innovation effort made by the manufacturer Green innovation effort made by the retailer Retail price Collecting rate Wholesale price
**Functions**	**Descriptions**
q $\pi_{m}$ $\pi_{r}$ $\pi_{c}$ $\pi_{sc}$	Demand for the product Manufacturer’s profit Retailer’s profit Collector’s profit Supply chain profit ($\pi_{sc}=\pi_{m}+\pi_{r}+\pi_{c}$)

**Table 2:** Six Stackelberg game models.

	Leader	Manufacturer-Led	Retailer-Led
Green Innovation			
**Manufacturer-driven** **Retailer-driven** **Both-driven**		*MM* *RM* *BM*	*MR* *RR* *BR*

## Appendix A

**Proof of Proposition** **1.** The second-order conditions (SOCs) of the retailer and the collector are expressed as $\partial^{2}\pi_{r}/\partial p^{2}=-2<0$ and $\partial^{2}\pi_{c}/\partial t^{2}=-\mu <0$, respectively. Thus, solving the first-order conditions (FOCs) of the retailer’s and collector’s problems leads to $t=(1-w+e_{m})(\rho -\alpha )/2\mu$ and $p=(1+w+e_{m})/2$, respectively. Integrating p and t into the manufacturer’s problem, we obtain the following Hessian matrix:

(A1) $$H_{m}^{MM}=\left(\begin{array}{cc} \frac{\partial^{2}\pi_{m}}{\partial w^{2}} & \frac{\partial^{2}\pi_{m}}{\partial w\partial e_{m}}\\ \frac{\partial^{2}\pi_{m}}{\partial e_{m}\partial w} & \frac{\partial^{2}\pi_{m}}{\partial e_{m}{}^{2}} \end{array}\right)=\left(\begin{array}{cc} -1+\frac{K_{1}}{2\mu } & \frac{1}{2}\left(1+\beta -\frac{K_{1}}{\mu }\right)\\ \frac{1}{2}\left(1+\beta -\frac{K_{1}}{\mu }\right) & -\beta -\theta +\frac{K_{1}}{2\mu } \end{array}\right).$$

Define $\Delta_{k,i}^{j}$ as the leading principal minor of order k in $H_{i}^{j}$ for i∈{m,r} and j∈Γ. If the condition $2\theta (2\mu -K_{1})>\mu {\left(1-\beta \right)}^{2}$ is met, we then have that $\Delta_{1,m}^{MM}<0$ and $\Delta_{2,m}^{MM}>0$, which implies that the manufacturer’s profit is strictly concave with respect to (w.r.t.) w and $e_{m}$. By solving the FOCs of the manufacturer’s problem, the equilibrium decisions and profits in Model *MM* are determined, as presented in Equation (3). This completes the proof. □

**Proof of Proposition** **2.** Define g=p−w>0 as the retailer’s marginal profit. The demand function is then rewritten as $q=1-w-g+e_{m}$. The SOC of the collector’s problem is given by $\partial^{2}\pi_{c}/\partial t^{2}=-\mu <0$. The Hessian matrix of the manufacturer’s objective function is given by

(A2) $$H_{m}^{MR}=\left(\begin{array}{cc} \frac{\partial^{2}\pi_{m}}{\partial w^{2}} & \frac{\partial^{2}\pi_{m}}{\partial w\partial e_{m}}\\ \frac{\partial^{2}\pi_{m}}{\partial e_{m}\partial w} & \frac{\partial^{2}\pi_{m}}{\partial e_{m}{}^{2}} \end{array}\right)=\left(\begin{array}{cc} -2 & 1+\beta \\ 1+\beta & -2\beta -\theta \end{array}\right).$$

We then find that $\Delta_{1,m}^{MR}<0$ and $\Delta_{2,m}^{MR}=2\theta -{\left(1-\beta \right)}^{2}$. If the condition $2\theta >{\left(1-\beta \right)}^{2}$ is met, we have $\Delta_{2,m}^{MR}>0$, implying that the manufacturer’s profit is strictly concave w.r.t. w and $e_{m}$. By solving the FOCs of the manufacturer’s and collector’s problems, we have

(A3) $$w=\frac{(1-g)\left(\beta \mu (1-\beta )+\theta (\mu -K_{1})\right)}{\theta (2\mu -K_{1})-\mu {\left(1-\beta \right)}^{2}},e_{m}=\frac{\mu (1-g)(1-\beta )}{\theta (2\mu -K_{1})-\mu {\left(1-\beta \right)}^{2}},\;\mathrm{and}\;t=\frac{\theta (1-g)(\rho -\alpha )}{\theta (2\mu -K_{1})-\mu {\left(1-\beta \right)}^{2}}.$$

Integrating w, $e_{m}$, and t in Equation (A3) into the retailer’s profit function, the SOC of the retailer’s problem is given by $\partial^{2}\pi_{r}/\partial g^{2}=-2\theta \mu /\left(\theta (2\mu -K_{1})-\mu {\left(1-\beta \right)}^{2}\right)$. We assume $\theta (2\mu -K_{1})>\mu {\left(1-\beta \right)}^{2}$ for optimality of the retailer’s problem; i.e., $\partial^{2}\pi_{r}/\partial g^{2}<0$. Solving the retailer’s FOC leads to g=1/2, which derives the equilibrium values in Equation (4). This completes the proof. □

**Proof of Corollary** **1.** From Propositions 1 and 2, we have the following relationships:

(A4) $$e_{m}^{MR}-e_{m}^{MM}=\frac{\mu^{2}{\left(1-\beta \right)}^{3}}{A_{1}A_{2}},t^{MR}-t^{MM}=\frac{\theta \mu {\left(1-\beta \right)}^{2}(\rho -\alpha )}{A_{1}A_{2}},\;\mathrm{and}\;q^{MR}-q^{MM}=\frac{\theta \mu^{2}{\left(1-\beta \right)}^{2}}{A_{1}A_{2}},$$

where $A_{1}=2\theta (2\mu -K_{1})-\mu {\left(1-\beta \right)}^{2}$ and $A_{2}=2\theta (2\mu -K_{1})-2\mu {\left(1-\beta \right)}^{2}$. Because $\theta (2\mu -K_{1})>\mu {\left(1-\beta \right)}^{2}$ holds, we have $A_{2}>A_{1}>0$, which implies that $e_{m}^{MR}>e_{m}^{MM}$, $t^{MR}>t^{MM}$, and $q^{MR}>q^{MM}$. This completes the proof. □

**Proof of Corollary** **2.** From Propositions 1 and 2, we have the following relationships:

(A5) $$\pi_{m}^{MR}-\pi_{m}^{MM}=\frac{\theta \mu \left(\mu A_{1}\left(2\theta -{\left(1-\beta \right)}^{2}\right)-A_{2}^{2}\right)}{2A_{1}A_{2}^{2}},\pi_{r}^{MR}-\pi_{r}^{MM}=\frac{\theta \mu (A_{1}^{2}-2\theta \mu A_{2})}{2A_{1}^{2}A_{2}},\;\mathrm{and}\;\;\pi_{c}^{MR}-\pi_{c}^{MM}=\frac{\theta^{2}\mu {\left(\rho -\alpha \right)}^{2}(A_{1}^{2}-A_{2}^{2})}{2A_{1}^{2}A_{2}^{2}}.$$

Given that $A_{1}>A_{2}>0$, $A_{1}^{2}>A_{2}^{2}$ and $\pi_{c}^{MR}>\pi_{c}^{MM}$. Due to the assumption that $\mu >K_{1}$, it is always true that $A_{1}^{2}>2\theta \mu A_{2}$, which leads to the inequality: $\pi_{r}^{MR}>\pi_{r}^{MM}$. If $\theta <K_{2}$, then $\mu A_{1}\left(2\theta -{\left(1-\beta \right)}^{2}\right)>A_{2}^{2}$; otherwise, $\mu A_{1}\left(2\theta -{\left(1-\beta \right)}^{2}\right)<A_{2}^{2}$, where

(A6) $$K_{2}=\frac{\mu {\left(1-\beta \right)}^{2}}{4}\left(\frac{5\mu -3K_{1}+\sqrt{\mu^{2}+6\mu K_{1}-3K_{1}^{2}}}{\left(\mu -K_{1})(2\mu -K_{1}\right)}\right).$$

Therefore, if $\theta <K_{2}$, then $\pi_{m}^{MR}>\pi_{m}^{MM}$; otherwise, $\pi_{m}^{MR}<\pi_{m}^{MM}$. This completes the proof. □

**Proof of Proposition** **3.** The SOC of the collector’s problem is expressed as $\partial^{2}\pi_{c}/\partial t^{2}=-\mu <0$. The Hessian matrix of the retailer’s problem is as follows:

(A7) $$H_{r}^{RM}=\left(\begin{array}{cc} \frac{\partial^{2}\pi_{r}}{\partial p^{2}} & \frac{\partial^{2}\pi_{r}}{\partial p\partial e_{r}}\\ \frac{\partial^{2}\pi_{r}}{\partial e_{r}\partial p} & \frac{\partial^{2}\pi_{r}}{\partial e_{r}{}^{2}} \end{array}\right)=\left(\begin{array}{cc} -2 & 1+\beta \\ 1+\beta & -2\beta -\theta \end{array}\right).$$

Assuming that $2\theta >{\left(1-\beta \right)}^{2}$, we can have that $\Delta_{1,r}^{RM}<0$ and $\Delta_{2,r}^{RM}>0$, implying that the retailer’s profit is strictly concave w.r.t. p and $e_{r}$. Solving the FOCs of the retailer’s and collector’s problems yields:

(A8) $$p=\frac{(\beta +\theta )(1+w)-w-\beta^{2}}{2\theta -{\left(1-\beta \right)}^{2}},e_{r}=\frac{\left(1-w)(1-\beta \right)}{2\theta -{\left(1-\beta \right)}^{2}},\;\mathrm{and}\;t=\frac{\theta (1-w)(\rho -\alpha )}{2\theta \mu -\mu {\left(1-\beta \right)}^{2}}.$$

Substituting p, $e_{r}$, and t in Equation (A8) into the manufacturer’s profit function, the SOC of the manufacturer’s problem is given by $\partial^{2}\pi_{m}/\partial w^{2}=-2\theta \left(\theta (2\mu -K_{1})-\mu {\left(1-\beta \right)}^{2}\right)/\mu {\left(2\theta -{\left(1-\beta \right)}^{2}\right)}^{2}$. We also assume $\theta (2\mu -K_{1})>\mu {\left(1-\beta \right)}^{2}$ for optimality of the manufacturer’s problem; i.e., $\partial^{2}\pi_{m}/\partial w^{2}<0$. Solving the manufacturer’s FOC leads to the equilibrium values in Equation (6). This completes the proof. □

**Proof of Proposition** **4.** Substituting g into the demand function, we have that $q=1-w-g+e_{r}$. The SOCs of the manufacturer’s and collector’s problems are given by $\partial^{2}\pi_{m}/\partial w^{2}=-2<0$ and $\partial^{2}\pi_{c}/\partial t^{2}=-\mu <0$. Therefore, solving the FOCs of the manufacturer’s and collector’s problems results in:

(A9) $$w=\frac{\left(\mu -K_{1})(1-g+e_{r}\right)}{2\mu -K_{1}}\;and\;t=\frac{\left(\rho -\alpha )(1-g+e_{r}\right)}{2\mu -K_{1}}.$$

We then integrate w and t in Equation (A9) into the retailer’s profit function and obtain the following Hessian matrix:

(A10) $$H_{r}^{RR}=\left(\begin{array}{cc} \frac{\partial^{2}\pi_{r}}{\partial g^{2}} & \frac{\partial^{2}\pi_{r}}{\partial g\partial e_{r}}\\ \frac{\partial^{2}\pi_{r}}{\partial e_{r}\partial g} & \frac{\partial^{2}\pi_{r}}{\partial e_{r}{}^{2}} \end{array}\right)=\left(\begin{array}{cc} -\frac{2\mu }{2\mu -K_{1}} & \frac{\mu (1+\beta )}{2\mu -K_{1}}\\ \frac{\mu (1+\beta )}{2\mu -K_{1}} & -\theta -\frac{2\beta \mu }{2\mu -K_{1}} \end{array}\right).$$

Assuming that $2\theta (2\mu -K_{1})>\mu {\left(1-\beta \right)}^{2}$, we can have that $\Delta_{1,r}^{RR}<0$ and $\Delta_{2,r}^{RR}>0$, implying that the retailer’s profit is strictly concave w.r.t. g and $e_{r}$. Solving the FOCs of the retailer’s problem yields

(A11) $$g=\frac{\theta (2\mu -K_{1})+\beta \mu (1-\beta )}{2\theta (2\mu -K_{1})-\mu {\left(1-\beta \right)}^{2}}\;and\;e_{r}=\frac{\mu (1-\beta )}{2\theta (2\mu -K_{1})-\mu {\left(1-\beta \right)}^{2}},$$

which results in the equilibrium values in Equation (7). This completes the proof. □

**Proof of Corollary** **3.** As the proof of Corollary 3 is similar to that of Corollary 1, we omit it here. □

**Proof of Corollary** **4.** As the proof of Corollary 4 is similar to that of Corollary 2, we omit it here. □

**Proof of Proposition** **5.** The SOC of the collector’s problem is given by $\partial^{2}\pi_{c}/\partial t^{2}=-\mu <0$. The Hessian matrix of the retailer’s problem is as follows:

(A12) $$H_{r}^{BM}=\left(\begin{array}{cc} \frac{\partial^{2}\pi_{r}}{\partial p^{2}} & \frac{\partial^{2}\pi_{r}}{\partial p\partial e_{r}}\\ \frac{\partial^{2}\pi_{r}}{\partial e_{r}\partial p} & \frac{\partial^{2}\pi_{r}}{\partial e_{r}{}^{2}} \end{array}\right)=\left(\begin{array}{cc} -2 & 1+\beta \\ 1+\beta & -2\beta -\theta \end{array}\right).$$

Assuming that $2\theta >{\left(1-\beta \right)}^{2}$, we have that $\Delta_{1,r}^{BM}<0$ and $\Delta_{2,r}^{BM}>0$, implying that the retailer’s profit is strictly concave w.r.t. p and $e_{r}$. Solving the FOCs of the retailer’s and collector’s problems yields:

(A13) $$p=\frac{\left(\beta -\beta^{2}+\theta )(1+e_{m})-w(1-\beta -\theta \right)}{2\theta -{\left(1-\beta \right)}^{2}},e_{r}=\frac{\left(1-w+e_{m})(1-\beta \right)}{2\theta -{\left(1-\beta \right)}^{2}},\;\mathrm{and}\;t=\frac{\theta (1-w+e_{m})(\rho -\alpha )}{2\theta \mu -\mu {\left(1-\beta \right)}^{2}}.$$

We then integrate p, $e_{r}$, and t in Equation (A13) into the manufacturer’s profit function and obtain the following Hessian matrix:

(A14) $$H_{m}^{BM}=\left(\begin{array}{cc} \frac{\partial^{2}\pi_{m}}{\partial w^{2}} & \frac{\partial^{2}\pi_{m}}{\partial w\partial e_{m}}\\ \frac{\partial^{2}\pi_{m}}{\partial e_{m}\partial w} & \frac{\partial^{2}\pi_{m}}{\partial e_{m}{}^{2}} \end{array}\right)=\left(\begin{array}{cc} -\frac{2\theta \left(\theta (2\mu -K_{1})-\mu {\left(1-\beta \right)}^{2}\right)}{\mu {\left(2\theta -{\left(1-\beta \right)}^{2}\right)}^{2}} & \frac{\theta \left(2\theta (\mu +\beta \mu -K_{1})-\mu (1+\beta ){\left(1-\beta \right)}^{2}\right)}{\mu {\left(2\theta -{\left(1-\beta \right)}^{2}\right)}^{2}}\\ \frac{\theta \left(2\theta (\mu +\beta \mu -K_{1})-\mu (1+\beta ){\left(1-\beta \right)}^{2}\right)}{\mu {\left(2\theta -{\left(1-\beta \right)}^{2}\right)}^{2}} & \frac{\theta \left(\mu (1-4\beta +\beta^{2}-2\theta )\left(2\theta -{\left(1-\beta \right)}^{2}\right)-2\theta K_{1}\right)}{\mu {\left(2\theta -{\left(1-\beta \right)}^{2}\right)}^{2}} \end{array}\right).$$

If $\theta (2\mu -K_{1})>\mu {\left(1-\beta \right)}^{2}$ and $2\theta (2\mu -K_{1})>3\mu {\left(1-\beta \right)}^{2}$, then $\Delta_{1,m}^{BM}<0$ and $\Delta_{2,m}^{BM}>0$, implying that the manufacturer’s profit is strictly concave w.r.t. w and $e_{m}$. Solving the FOCs of the manufacturer’s problem yields

(A15) $$w=\frac{2\theta (\mu -K_{1})-\mu (1-\beta )(1-2\beta )}{2\theta (2\mu -K_{1})-3\mu {\left(1-\beta \right)}^{2}}\;and\;e_{m}=\frac{\mu (1-\beta )}{2\theta (2\mu -K_{1})-3\mu {\left(1-\beta \right)}^{2}},$$

which results in the equilibrium values in Equation (9). This completes the proof. □

**Proof of Proposition** **6.** We substitute g into the demand function, leading to $q=1-w-g+e_{m}+e_{r}$. The SOC of the collector’s problem is given by $\partial^{2}\pi_{c}/\partial t^{2}=-\mu <0$. The Hessian matrix of the manufacturer’s problem is shown below.

(A16) $$H_{m}^{BR}=\left(\begin{array}{cc} \frac{\partial^{2}\pi_{m}}{\partial w^{2}} & \frac{\partial^{2}\pi_{m}}{\partial w\partial e_{m}}\\ \frac{\partial^{2}\pi_{m}}{\partial e_{m}\partial w} & \frac{\partial^{2}\pi_{m}}{\partial e_{m}{}^{2}} \end{array}\right)=\left(\begin{array}{cc} -2 & 1+\beta \\ 1+\beta & -2\beta -\theta \end{array}\right).$$

Assuming that $2\theta >{\left(1-\beta \right)}^{2}$, we have $\Delta_{1,m}^{BR}<0$ and $\Delta_{2,m}^{BR}>0$, implying that the manufacturer’s profit is strictly concave w.r.t. w and $e_{m}$. Solving the FOCs of the manufacturer’s and collector’s problems yields:

(A17) $$w=\frac{(1-g+e_{r})\left(\theta (\mu -K_{1})+\mu \beta (1-\beta )\right)}{\theta (2\mu -K_{1})-\mu {\left(1-\beta \right)}^{2}},e_{m}=\frac{\mu (1-\beta )(1-g+e_{r})}{\theta (2\mu -K_{1})-\mu {\left(1-\beta \right)}^{2}},\;\mathrm{and}\;t=\frac{\theta (\rho -\alpha )(1-g+e_{r})}{\theta (2\mu -K_{1})-\mu {\left(1-\beta \right)}^{2}}.$$

We then integrate w, $e_{m}$, and t in Equation (A17) into the retailer’s profit function and obtain the following Hessian matrix:

(A18) $$H_{r}^{BR}=\left(\begin{array}{cc} \frac{\partial^{2}\pi_{r}}{\partial g^{2}} & \frac{\partial^{2}\pi_{r}}{\partial g\partial e_{r}}\\ \frac{\partial^{2}\pi_{r}}{\partial e_{r}\partial g} & \frac{\partial^{2}\pi_{r}}{\partial e_{r}{}^{2}} \end{array}\right)=\left(\begin{array}{cc} -\frac{2\theta \mu }{\theta (2\mu -K_{1})-\mu {\left(1-\beta \right)}^{2}} & \frac{\theta \mu (1+\beta )}{\theta (2\mu -K_{1})-\mu {\left(1-\beta \right)}^{2}}\\ \frac{\theta \mu (1+\beta )}{\theta (2\mu -K_{1})-\mu {\left(1-\beta \right)}^{2}} & -\theta -\frac{2\beta \theta \mu }{\theta (2\mu -K_{1})-\mu {\left(1-\beta \right)}^{2}} \end{array}\right).$$

If $\theta (2\mu -K_{1})>\mu {\left(1-\beta \right)}^{2}$ and $2\theta (2\mu -K_{1})>3\mu {\left(1-\beta \right)}^{2}$, then $\Delta_{1,r}^{BR}<0$ and $\Delta_{2,r}^{BR}>0$, implying that the retailer’s profit is strictly concave w.r.t. g and $e_{r}$. Solving the FOCs of the retailer’s problem yields

(A19) $$g=\frac{1}{2}\left(1+\frac{\mu (1-\beta^{2})}{2\theta (2\mu -K_{1})-3\mu {\left(1-\beta \right)}^{2}}\right)\;and\;e_{r}=\frac{\mu (1-\beta )}{2\theta (2\mu -K_{1})-3\mu {\left(1-\beta \right)}^{2}},$$

which results in the equilibrium values in Equation (10). This completes the proof. □

**Proof of Corollary** **5.** From Propositions 5 and 6, Corollary 5 is true. This completes the proof. □

**Proof of Corollary** **6.** From Propositions 5 and 6, we have that

(A20) $$\pi_{m}^{BM}-\pi_{m}^{BR}=\pi_{r}^{BR}-\pi_{r}^{BM}=\frac{\theta \mu \left(\theta (\mu -K_{1})-\mu {\left(1-\beta \right)}^{2}\right)}{{\left(2\theta (2\mu -K_{1})-3\mu {\left(1-\beta \right)}^{2}\right)}^{2}}.$$

If $\theta (\mu -K_{1})>\mu {\left(1-\beta \right)}^{2}$, then $\pi_{m}^{BM}>\pi_{m}^{BR}$ and $\pi_{r}^{BM}<\pi_{r}^{BR}$; otherwise, $\pi_{m}^{BM}<\pi_{m}^{BR}$ and $\pi_{r}^{BM}>\pi_{r}^{BR}$. This completes the proof. □

## Appendix B

**Proof of Corollary** **7.** From Propositions 1–6, we have that $e_{t}^{BM}=e_{t}^{BR}$, $e_{t}^{MR}=e_{t}^{RM}$, $e_{t}^{MM}=e_{t}^{RR}$, $t^{BM}=t^{BR}$, $t^{MR}=t^{RM}$, $t^{MM}=t^{RR}$, $q^{BM}=q^{BR}$, $q^{MR}=q^{RM}$, and $q^{MM}=q^{RR}$. In addition, we have the following relationships:

(A21) $$e_{t}^{BR}-e_{t}^{MR}=\frac{\mu (1-\beta )A_{1}}{A_{2}A_{3}}>0,e_{t}^{RM}-e_{t}^{MM}=\frac{\mu^{2}{\left(1-\beta \right)}^{3}}{A_{1}A_{2}}>0,t^{BR}-t^{MR}=\frac{\theta \mu (\rho -\alpha ){\left(1-\beta \right)}^{2}}{A_{2}A_{3}}>0,\;t^{RM}-t^{MM}=\frac{\theta \mu (\rho -\alpha ){\left(1-\beta \right)}^{2}}{A_{1}A_{2}}>0,q^{BR}-q^{MR}=\frac{\theta \mu^{2}{\left(1-\beta \right)}^{2}}{A_{2}A_{3}}>0,\;\mathrm{and}\;q^{RM}-q^{MM}=\frac{\theta \mu^{2}{\left(1-\beta \right)}^{2}}{A_{1}A_{2}}>0,$$

where $A_{3}=2\theta (2\mu -K_{1})-3\mu {\left(1-\beta \right)}^{2}>0$. This completes the proof. □

**Proof of Corollary** **8.** From Propositions 1, 3, and 5, we have the following relationships:

(A22) $$\pi_{m}^{RM}-\pi_{m}^{MM}=\frac{\theta \mu^{2}{\left(1-\beta \right)}^{2}}{2A_{1}A_{2}}>0,\pi_{m}^{BM}-\pi_{m}^{RM}=\frac{\theta \mu^{2}{\left(1-\beta \right)}^{2}}{2A_{2}A_{3}}>0,\;\pi_{r}^{RM}-\pi_{r}^{MM}=\frac{\theta \mu^{2}{\left(1-\beta \right)}^{2}\left(2\theta K_{1}A_{2}+\mu^{2}{\left(1-\beta \right)}^{2}\left(2\theta -{\left(1-\beta \right)}^{2}\right)\right)}{2A_{1}^{2}A_{2}^{2}}>0,\;\pi_{r}^{BM}-\pi_{r}^{RM}=\frac{\theta \mu^{2}(A_{2}^{2}-A_{3}^{2})\left(2\theta -{\left(1-\beta \right)}^{2}\right)}{2A_{2}^{2}A_{3}^{2}}>0,\pi_{c}^{RM}-\pi_{c}^{MM}=\frac{\theta^{2}\mu (\rho -\alpha )(A_{1}^{2}-A_{2}^{2})}{2A_{1}^{2}A_{2}^{2}}>0,\mathrm{and}\;\pi_{c}^{BM}-\pi_{c}^{RM}=\frac{\theta^{2}\mu (\rho -\alpha )(A_{2}^{2}-A_{3}^{2})}{2A_{2}^{2}A_{3}^{2}}>0$$

Because $A_{1}>A_{2}>A_{3}>0$, it is true that $A_{1}^{2}>A_{2}^{2}>A_{3}^{2}>0$. This completes the proof. □

**Proof of Corollary** **9.** As the proof of Corollary 9 is similar to that of Corollary 8, we omit it here. □

**Proof of Corollary** **10.** From Propositions 1–4, we have that

(A23) $$\pi_{m}^{MR}-\pi_{m}^{RM}=\frac{\theta \mu (2\theta \mu -A_{1})}{2A_{2}^{2}}\;and\;\pi_{m}^{MM}-\pi_{m}^{RR}=\frac{\theta \mu (A_{1}-2\theta \mu )}{2A_{1}^{2}}.$$

If $\theta <K_{3}/2$, then $2\theta \mu >A_{1}$ and $\pi_{m}^{MR}>\pi_{m}^{RM}$ and $\pi_{m}^{MM}<\pi_{m}^{RR}$; otherwise, $\pi_{m}^{MR}<\pi_{m}^{RM}$ and $\pi_{m}^{MM}>\pi_{m}^{RR}$. We already know, from Corollaries 8 and 9, that $\pi_{m}^{MR}>\pi_{m}^{RR}$ and $\pi_{m}^{RM}>\pi_{m}^{MM}$. Thus, if $\theta <K_{3}/2$, we have $\pi_{m}^{MR}>\pi_{m}^{RM}>\pi_{m}^{MM}$, $\pi_{m}^{MR}>\pi_{m}^{RR}$, $\pi_{m}^{MM}<\pi_{m}^{RR}<\pi_{m}^{MR}$, and $\pi_{m}^{MM}<\pi_{m}^{RM}$, which implies that $\pi_{m}^{MR}>\max\left\{\pi_{m}^{RM},\pi_{m}^{MM},\pi_{m}^{RR}\right\}$ and $\pi_{m}^{MM}<\min\left\{\pi_{m}^{MR},\pi_{m}^{RM},\pi_{m}^{RR}\right\}$. If $\theta >K_{3}/2$, we have $\pi_{m}^{RM}>\pi_{m}^{MR}>\pi_{m}^{RR}$, $\pi_{m}^{RM}>\pi_{m}^{MM}$, $\pi_{m}^{RR}<\pi_{m}^{MM}<\pi_{m}^{RM}$, and $\pi_{m}^{RR}<\pi_{m}^{MR}$, which implies that $\pi_{m}^{RM}>\max\left\{\pi_{m}^{MR},\pi_{m}^{MM},\pi_{m}^{RR}\right\}$ and $\pi_{m}^{RR}<\min\left\{\pi_{m}^{MR},\pi_{m}^{RM},\pi_{m}^{MM}\right\}$. For the retailer, the same argument holds; thus the proof is omitted here. We can find, from Propositions 1–4, that $\pi_{c}^{MR}=\pi_{c}^{RM}$ and $\pi_{c}^{MM}=\pi_{c}^{RR}$, and, from Corollaries 2 and 4, that $\pi_{c}^{MR}>\pi_{c}^{MM}$ and $\pi_{c}^{RM}>\pi_{c}^{RR}$. This leads to $\pi_{c}^{MR}=\pi_{c}^{RM}>\pi_{c}^{MM}=\pi_{c}^{RR}$, which completes the proof. □

**Proof of Corollary** **11.** Because $\theta >K_{4}>K_{3}/2$, we have $\pi_{m}^{RM}>\max\left\{\pi_{m}^{MR},\pi_{m}^{MM},\pi_{m}^{RR}\right\}$ from Corollary 10. If $K_{4}<\theta <K_{3}$, then $\pi_{m}^{BR}>\pi_{m}^{BM}>\pi_{m}^{RM}$ from Corollaries 6 and 8, indicating that $\pi_{m}^{BR}>\max\left\{\pi_{m}^{BM},\pi_{m}^{RM},\pi_{m}^{MR},\pi_{m}^{MM},\pi_{m}^{RR}\right\}$. If $\theta >K_{3}$, we still have $\pi_{m}^{RM}>\max\left\{\pi_{m}^{MR},\pi_{m}^{MM},\pi_{m}^{RR}\right\}$, $\pi_{m}^{BM}>\pi_{m}^{BR}$, and $\pi_{m}^{BM}>\pi_{m}^{RM}$, which leads to the inequality $\pi_{m}^{BM}>\max\left\{\pi_{m}^{BR},\pi_{m}^{RM},\pi_{m}^{MR},\pi_{m}^{MM},\pi_{m}^{RR}\right\}$. If $\theta >K_{4}$, we have $\pi_{m}^{RR}<\min\left\{\pi_{m}^{MR},\pi_{m}^{RM},\pi_{m}^{MM}\right\}$ from Corollary 10. We already know that $\pi_{m}^{RR}<\pi_{m}^{BR}$ and $\pi_{m}^{RR}<\pi_{m}^{MM}<\pi_{m}^{BM}$ from Corollaries 8 and 9. Therefore, $\pi_{m}^{RR}<\min\left\{\pi_{m}^{BM},\pi_{m}^{BR},\pi_{m}^{RM},\pi_{m}^{MR},\pi_{m}^{MM}\right\}$. For the retailer, the same argument holds; hence, we omit the proof here. It is easily found from Propositions 1-4 that $\pi_{c}^{BR}=\pi_{c}^{BM}$ and $\pi_{c}^{MR}=\pi_{c}^{RM}$. From Corollaries 8 and 9, we have $\pi_{c}^{BR}>\pi_{c}^{MR}$ and $\pi_{c}^{BM}>\pi_{c}^{RM}$. Using Corollary 10, it can be obtained that $\pi_{c}^{BM}=\pi_{c}^{BR}>\pi_{c}^{RM}=\pi_{c}^{MR}>\pi_{c}^{MM}=\pi_{c}^{RR}$. This completes the proof. □

**Proof of Corollary** **12.** From Propositions 1–4, we have

(A24) $$\pi_{sc}^{MM}=\pi_{sc}^{RR}=\frac{\theta \mu \left(A_{1}+\theta \left(2\mu +{\left(\rho -\alpha \right)}^{2}\right)\right)}{2A_{1}^{2}},\pi_{sc}^{RM}=\pi_{sc}^{MR}=\frac{\theta \mu \left(A_{3}+\theta \left(2\mu +{\left(\rho -\alpha \right)}^{2}\right)\right)}{2A_{2}^{2}},\;\mathrm{and}\;\;\pi_{sc}^{BM}=\pi_{sc}^{BR}=\frac{\theta \mu \left(A_{3}+\theta \left(2\mu +{\left(\rho -\alpha \right)}^{2}\right)-\mu {\left(1-\beta \right)}^{2}\right)}{2A_{3}^{2}}.$$

From Corollaries 8 and 9, we find that $\pi_{sc}^{BM}>\pi_{sc}^{RM}>\pi_{sc}^{MM}$ and $\pi_{sc}^{BR}>\pi_{sc}^{MR}>\pi_{sc}^{RR}$, leading to the conclusion that $\pi_{sc}^{BM}=\pi_{sc}^{BR}>\pi_{sc}^{RM}=\pi_{sc}^{MR}>\pi_{sc}^{MM}=\pi_{sc}^{RR}$. This completes the proof. □
